# A surgical epicardial approach to mapping and ablation in large animal models

**DOI:** 10.1007/s10840-026-02353-3

**Published:** 2026-05-21

**Authors:** Ken S. Lizama, Abdelmotogaly Elgalad, Tirth A. Patel, Matthews John, Mehdi Razavi, Mark D. McCauley

**Affiliations:** 1 Division of Cardiology, Department of Medicine, College of Medicine, University of Illinois Chicago, Chicago, IL USA; 2 Jesse Brown VA Medical Center, Chicago, IL USA; 3 Department of Physiology and Biophysics and the Center for Cardiovascular Research, College of Medicine, University of Illinois at Chicago, Chicago, IL USA; 4 Texas Heart Institute and Baylor College of Medicine, Houston, TX USA; 5 Division of Cardiology, Department of Medicine, University of Illinois Chicago, 840 S. Wood St., 920S (MC 715), Chicago, IL 60612 USA

**Keywords:** Epicardial, Large animal, Surgical models

## Abstract

Large animal surgeries are the standard for validating pre-clinical electrophysiology studies, and serve as an important step for the rationale underlying clinical trials. Although electrophysiologists receive extensive training in catheter-based approaches, few medical centers have the capability to perform experimental animal surgeries in large animals. Here, we present a step-by-step guide to performing an epicardial approach to pacing and sensing in a large animal sheep model, with the goal of enabling a wide variety of pacing and ablation options.

## Introduction

In experimental and translational cardiac electrophysiology, surgical epicardial access allows for the opportunity to test hypotheses with devices that directly interface with the epicardial surface of the heart. Often, open surgical techniques are a required first step in pre-clinical development of new catheters and procedures, and can facilitate: epicardial visualization, standardize surgical approaches, and can allow for experimental flexibility [1–5]. We and others have used surgical access in large animal models to create unique catheter arrangements, create radiofrequency scar shapes that promote anisotropy, and test experimental therapies, such as the application of carbon nanotube fibers (CNTf) to facilitate electrical conduction within the heart [6, 7]. While most cardiac electrophysiologists are facile at intracardiac catheter techniques, and many are able to access the epicardial space with catheter-based subxiphoid or transapical approaches, few have training and experience in thoracic surgical techniques to expose the epicardium. Therefore, in this experimental methods paper, we review the applications of surgical epicardial access and provide a detailed description of how we surgically access the epicardium. Considerations are given to management of the pericardium and EP catheter setup.

## Methods

### Materials

**Fig. 1:**
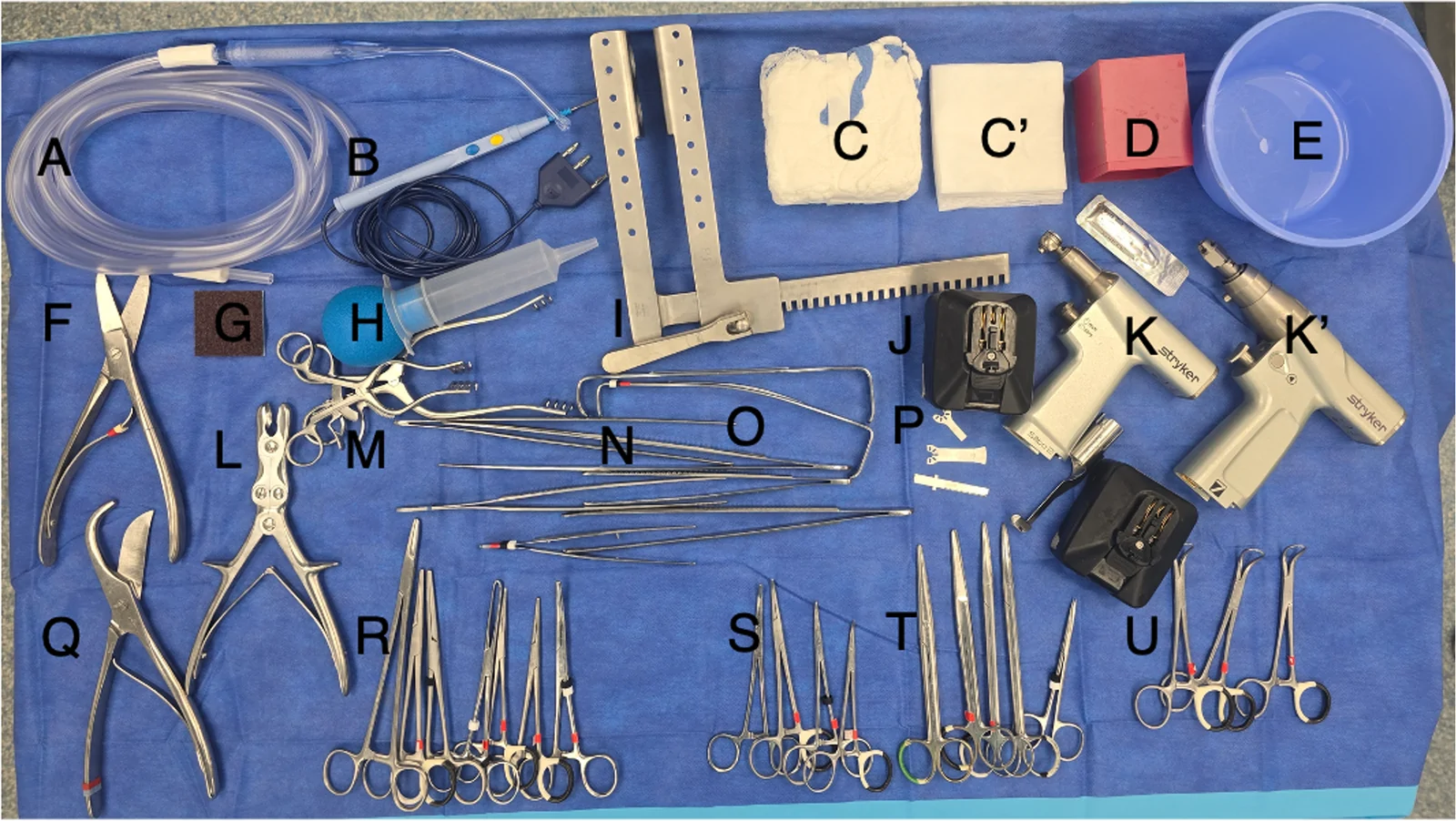
Overview of instruments used for sternotomy **Table:** 

A list of surgical tools and materials necessary to perform the surgeries is provided in Table 1. Also, Fig. 1 shows a typical surgical tray setup for the planned surgery.

**Table 1:** List of materials necessary for surgery

Animal Preparation	
Animal	For example, adult sheep 70 kg in our laboratory
Preparation Equipment	Electric shears or clippers; Depilatory cream (e.g., Nair); Laryngoscope; Endotracheal tube; IV catheter and line; Rumen tube
Anesthesia & Analgesia	
Ketamine	**Dosage**: 7.5 mg/kg bolus; 0.6–1.2 mg/kg/hr; **Route**: IV; **Frequency**: Peri-op (CRI); **Purpose**: Induction and maintenance
Midazolam	**Dosage**: 0.3 mg/kg; **Route**: IV; **Frequency**: Pre-op; **Purpose**: Sedation
Glycopyrrolate	**Dosage**: 0.01 mg/kg; **Route**: IV; **Frequency**: Pre-op; **Purpose**: Control secretions
Propofol	**Dosage**: 2–4 mg/kg; **Route**: IV; **Frequency**: To effect; **Purpose**: Intubation
Isoflurane	**Dosage**: 0.5–3% in O₂ (40–100%); **Route**: Inhaled; **Frequency**: Maintenance; **Purpose**: Anesthesia maintenance
Hydromorphone	**Dosage**: 0.05–0.1 mg/kg; **Route**: IV or IM; **Frequency**: Q2H or as needed; **Purpose**: Analgesia
Flunixin	**Dosage**: 2.2 mg/kg; **Route**: IV or IM; **Frequency**: Pre-/Post-op; **Purpose**: NSAID
Cefazolin	**Dosage**: 25 mg/kg; **Route**: IV; **Frequency**: Q2H (in survival surgeries); **Purpose**: Antibiotic
Surgical Instruments	
General Tools	Sterile surgical drapes; Marker pen; Silk sutures (3 − 0, 4 − 0); Prolene sutures (4 − 0, 5 − 0)
Retraction and Dissection	Hemostats; Finochietto rib spreader; Tissue retractors; Rib cutters; Forceps
Electrosurgical	Valleylab Force FXc electrosurgical generator; Bovie cautery (cut/coagulation modes)
Suction System	Suction kit; Suction tip; Tubing; Canister (800 cc)
EP Equipment	
Recording and Mapping	EGM recording system; 3D mapping system; Reference patches
Stimulation and Ablation	Pace stimulator; RF ablation generator and catheter; Irrigation pump and tubing
Catheters	Duodecapolar ring catheter; Connecting cables

Label	Instrument name	Primary use
A	Suction / evacuation tubing with Yankauer suction handle	Evacuating blood/fluids
B	Electrosurgical (Bovie) pencil with cord & two-button control	Cutting / coagulation
C (C’)	Laparotomy sponge & gauze sponge	High-capacity absorbing and general blotting
D	Sharps foam container	Safely dispose of used suture needles and other sharps
E	Irrigation basin	Holding solution
F	Liston bone-cutting forceps – straight	Heavy double-action bone cutter
G	Electrosurgical-tip scratch pad (Bovie scratch pad)	Cleans the Bovie tip
H	Bulb irrigator syringe (50 mL “Asepto” bulb)	Pulsed or bulb irrigation
I	Finochietto rib-retractor	Rib spreader
J	Sterile lithium battery packs for Stryker System	Power source for the stryker system
K (K’)	Stryker System 7 orthopaedic power tool	Cutting & drilling bone
L	Stille-Luer double-action rongeur	Nibbling bone edges
M	Weitlaner self-retaining retractors	Hold soft tissue apart
N	Forceps	Grasping fascia/soft tissue
O	Malleable (ribbon) retractors	Shape-able soft-tissue retractor
P	Replacement oscillating-saw blade	Spare saw for the stryker system
Q	Liston bone-cutting forceps – curved (rib shears)	Cutting ribs / curved bone
R	Needle holders	Drive curved suture needle
S	Curved forceps	Clamping and holding tissues or blood vessels
T	Straight Mayo scissors, metzenbaum	Cutting thick tissue / suture
U	Towel clamps	To secure drapes

### Animal preparation and intubation

In our laboratory we use sheep, due to the size of the sheep heart, which is similar to human patients. We typically use 65–80 kg, adult Q-fever tested Suffolk crossed sheep for research purposes. A similar approach may be used in pigs and other species. Sheep are shaved, prepped, and anesthetized according to standard surgical procedure, and according to university IACUC guidelines. Specifically, after shaving wool over the surgical site and apply depilatory cream to create an optimum skin to electrode contact, then reference electrode patches are placed and checked for contact. Electrode gels is also used to reduce the high impedance. Sheep is positioned supine, in the area of the surgical site ventrally, the chest is swabbed with betadine scrub followed by alcohol three times, followed by betadine solution paint, and a sterile surgical drape is placed over the surgical site.

### Anesthesia

We typically have a dedicated veterinary technician who administers anesthetic agents for each surgery. It is useful for us to review the anesthetic protocol with the technician prior to induction, given the complexity of the anesthetic regimen. Sheep are sedated with 7.5 mg/kg Ketamine IV + 0.3 mg/kg midazolam IV. Glycopyrolate 0.01 mg/kg, Propofol (2–4 mg/kg IV) is given to anesthetic effect to allow for intubation and for placement of a rumen tube. Flunixin 2.2 mg/kg IM is given preoperatively for intraoperative analgesia. Hydromorphone 0.05–0.1 mg/kg IV is given preoperatively for intraoperative analgesia and will be given Q2H or as needed for pain control. Anesthesia is maintained with isoflurane (0.5-3%). For animals receiving survival/chronic studies: Cefazolin (25 mg/kg IV) is given preoperatively and every 2 h during surgery. Rocuronium, a non-depolarizing neuromuscular blocker (0.04 − 0.10 mg/kg IV) is given prior to initial incision to minimize muscle spasm during the use of the bovie pen (Table 1).

### Sternotomy

**Fig. 2:**
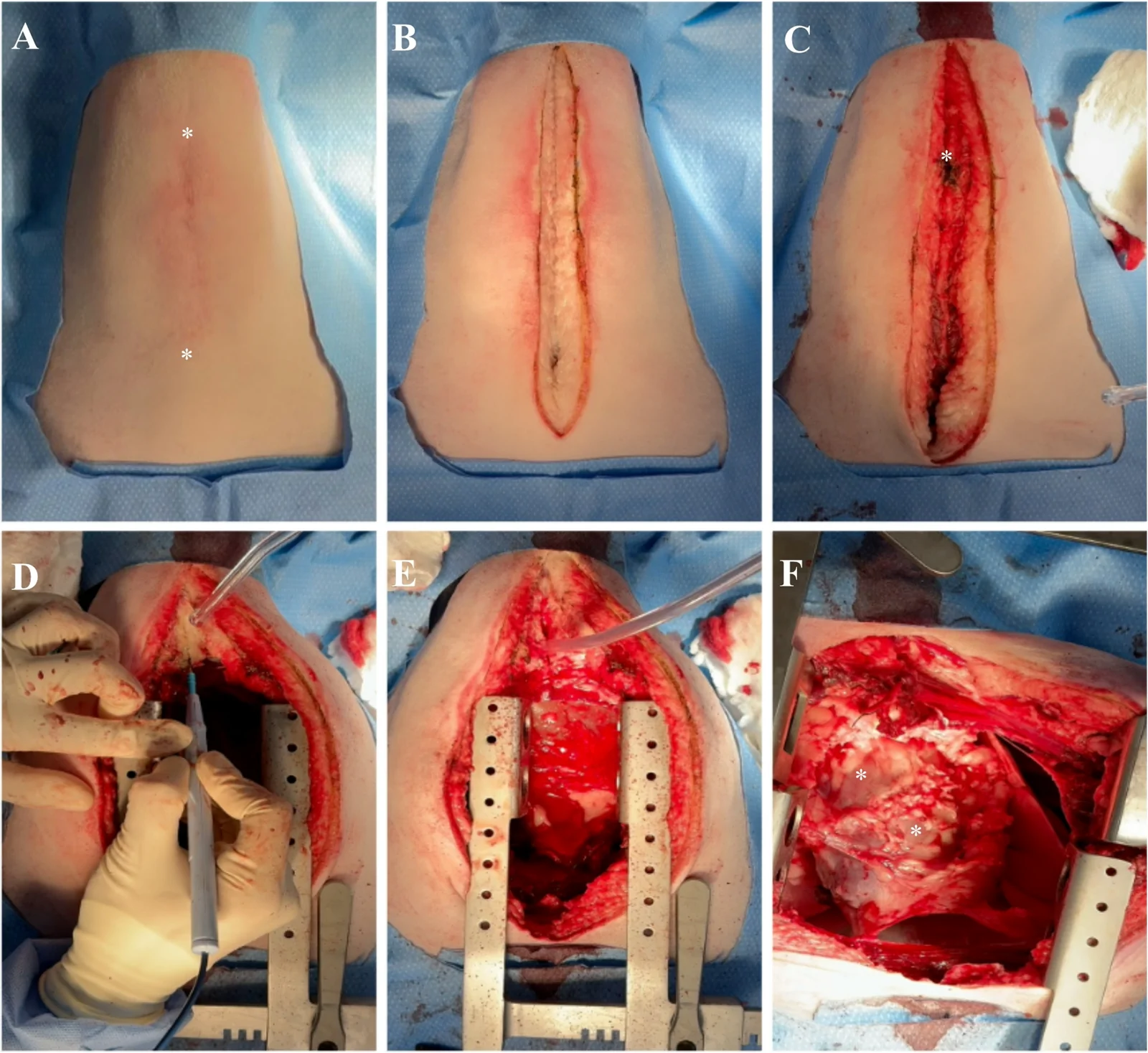
Sternotomy Steps. **A** Presurgical site covered with drape exposed obtain maximum view for sternotomy. ** Refer to the area between the manubrium superiorly and the xiphoid inferiorly. **B** Bovie (cauterization mode) is used to cut vertical midline incision of approximately 30 cm. **C** Bovie (coagulation mode) is used to dissect layers of fatty and subcutaneous tissue and then identify the sternal ligament. * Shows the location of a highly vascular fat layer. **D** and **E** Thorax is fully opened and retracted using Finochietto rib spreader. **F** Fully exposed cardiac tissue with intact pericardium. If needed, use additional spreader to expose the superior structures of the heart. *Right atrium, mostly of appendage and the left ventricle

Midsternal incision from the sternomanubrial joint to the xiphoid is made with low power cautery (15–25 W, cut setting). Consider carefully cauterizing the midline vertically about 30 cm. Carefully separate and cauterize through the skin, the epidermis and the dermis to expose the fatty layer (Fig. 2A and B). Once the initial cut is made, cautery/cut mode should be switched to coagulation mode with again minimum power (25–35 W, coagulation setting) necessary to incise and separate into the two layers of fatty and subcutaneous tissue, minimize bleeding at the thick hypodermis fat layer with small vessels. Feel with your finger to identify midline sternum and the sternal ligament by palpating, making sure that there is no incisional drifting from the midline (Fig. 2B and C). Identify the xiphoid process and make a small incision using the Metzenbaum (Table 1T) at the subxiphoid. Using your finger, carefully separate and swipe the pericardial fascia from the internal thoracic wall. Use a bone-cutting saw (Table 1K) to separate the sternum midline until you reach the manubrium; sometimes an oscillating saw is needed to complete the sternal division due to the thickness of the manubrium and the deep angle in animals compared to human anatomy (Fig. 2D). Thorax is fully opened and retracted using Finochietto rib spreader (Table 1). Fully expose the cardiac tissue with intact pericardium. If needed use additional retractor to expose the superior structures of the heart (Fig. 2E and F**)**.

### Pericardial resection

**Fig. 3:**
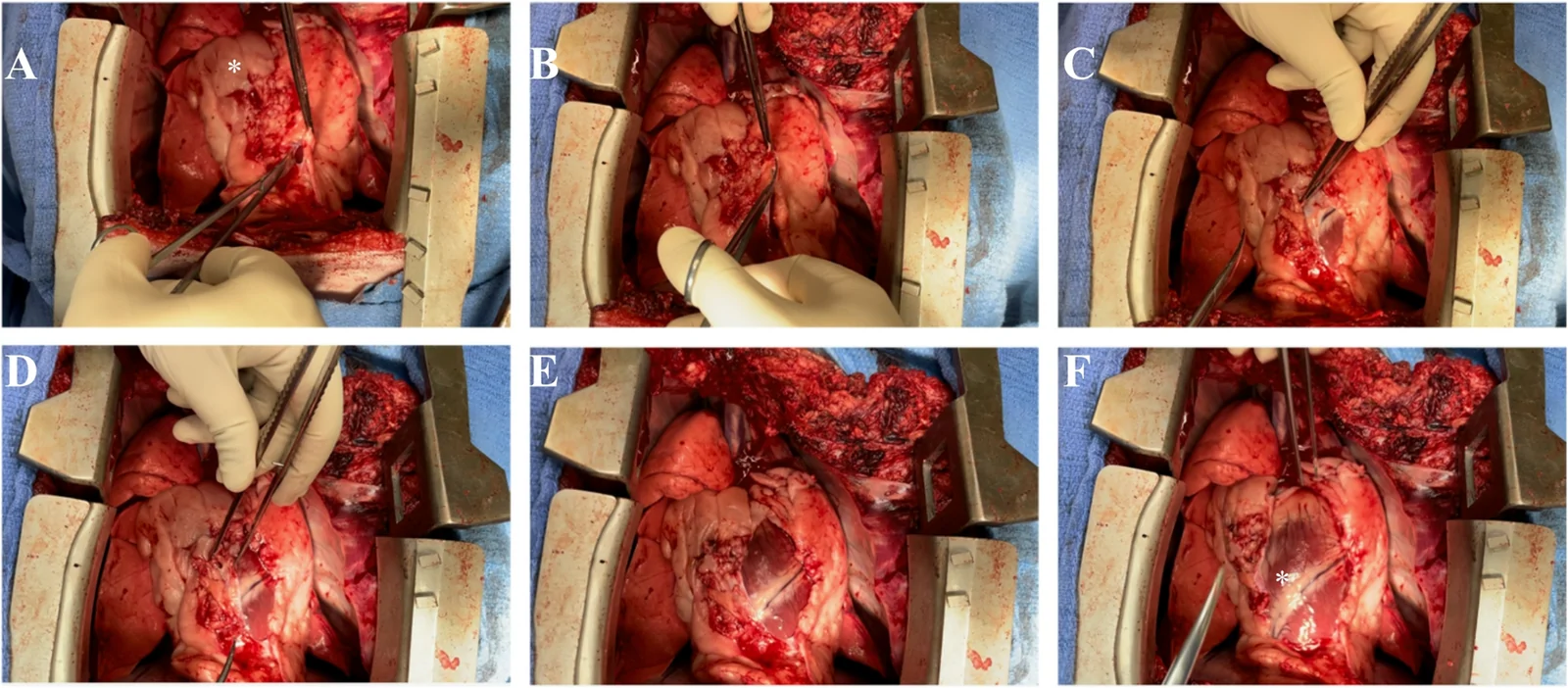
Resection of the Pericardium. **A** Lift the pericardium with forceps to separate up and away from the coronary arteries. *Thick layer of pericardial fat. **B** Make a midline incision to the pericardium using Metzenbaum scissors. **C** Extend the incision to the superior and inferior poles of the heart. **D** through **F** Peel back the pericardium to expose the left ventricle and left anterior descending coronary artery. *Left atrial descending vessels surrounded by epicardial fat

Identify the pericardium and the heart structures such as the appendages through the pericardium. Ovine pericardium has a vast amount of pericardial adipose tissue and may require resection of a layer to observe the heart anatomy. In resecting the pericardium, first lift the pericardium with tissue forceps to separate it up and away from the coronary arteries (Fig. 3A**)**. Using a Metzenbaum Scissor incise and cut bidirectionally above and below from slightly left of the midline pericardium, extending the incision to the superior and inferior poles of the heart (Fig. 3B-F**)**.

**Fig. 4:**
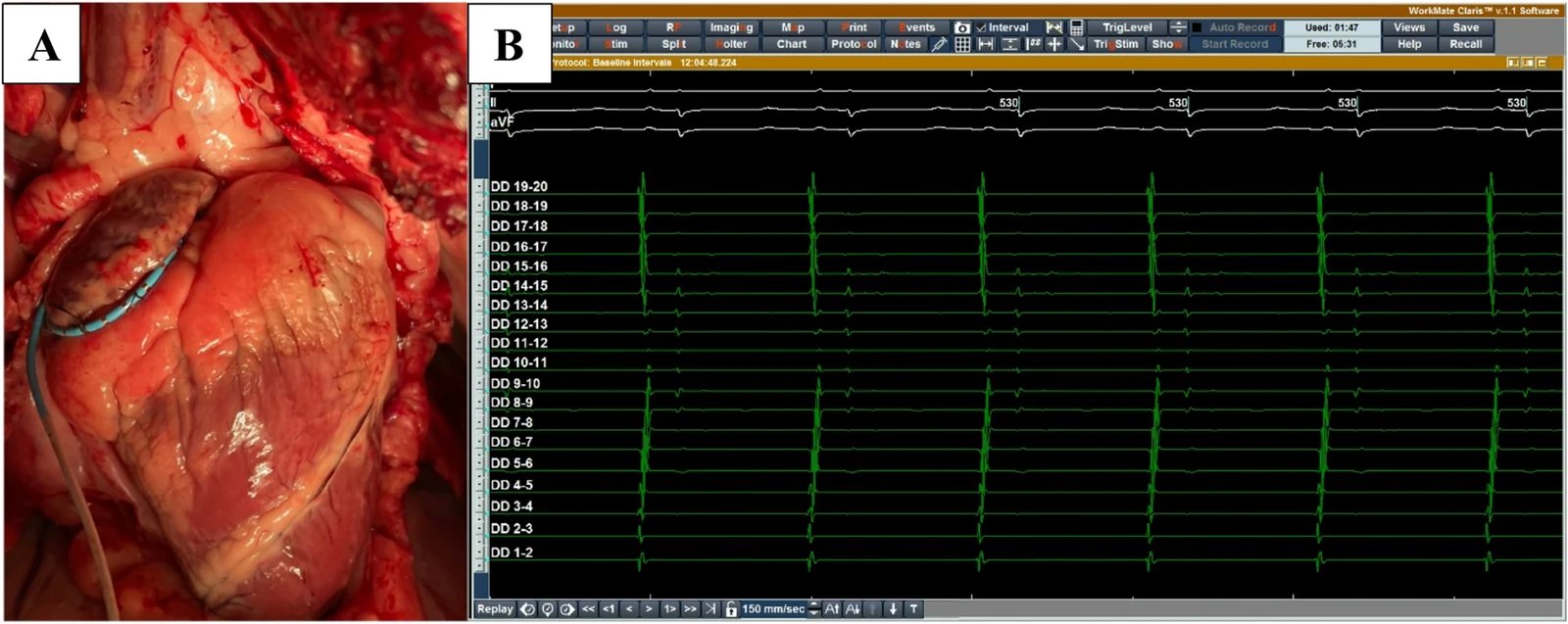
Recording of Atrial Electrograms from the Right Atrial Appendage. **A** A photograph of the sheep heart with a duodecapolar catheter encircling the right atrial appendage. **B** A recording of the electrograms taken from the St. Jude WorkMate Claris^TM^ recording system

The separated pericardium will be pulled at four corners positioned right and left superior and inferior site, to create a cradle to lift the heart and expose the area of epicardium of interest. 3 − 0 or 2 − 0 diameter size monofilament suture. Suture needle will pass through the thorax and clipped on both ends of the suture with a towel clamp to adjust according to the epicardial surface of interest (Fig. 4).

### Epicardial catheter setup and EP study

A variety of integrated recording, pacing, and mapping systems can be employed. In our lab, we use a St. Jude WorkMate Claris™ System and EP-4™ Cardiac Stimulator (Abbott Cardiovascular, Plymouth, MN) to record surface and intracardiac electrograms (EGM). For epicardial sensing and pacing we commonly use a 7 F 20-pole DD catheter (BLAZER™ DX-20, Boston Scientific, Boston MA). Additionally for radiofrequency ablation we use the INTELANAV MIFI™ catheter (Boston Scientific, Boston MA), supporting generator system (Maestro 4000™ Boston Scientific, Boston MA).

### Atrial study

The right atrial appendage (RAA) of sheep is readily accessible and has a good surface area to perform EP studies. The auricular shape of the appendage allows for favorable fitting of a circularly shaped DD catheter. For increased stability consider using stay stitches. The recording DD catheter must be positioned and fitted to minimize v-wave conduction. We typically use the EP Claris Workmate to record epicardial electrogram and deliver atrial pacing protocol such as atrial burst and extrastimulus pacing.

### Ventricular study

When performing ventricular preclinical studies, a defibrillator must be prepared and available. Ovine hearts are susceptible to ventricular fibrillation during catheter insertion or pacing protocols. Antiarrhythmic drugs may be prescribed during the course of the study if necessary, such as amiodarone or lidocaine. Surgical access provides exposure to both ventricles for epicardial EP studies. EP catheters, such as decapolar catheters, can be placed directly on the epicardium with one or more stitches along the length of the catheter to ensure good contact with the underlying myocardium. Since ovine hearts have significant epicardial fat pads compared to other animal hearts, care must be taken to place electrodes in direct contact with the myocardial tissue. Surgical epicardial pacing wires (Zimmer Biomet, Warsaw, IN) or metallic needle electrodes (Impulse Medical Technologies, Inc, Enumclaw, WA) for more controlled electrode placement. Once the electrodes are placed in their desired locations, EP data may be recorded using a compatible cable (or alligator clips in the case of electrodes not housed on a catheter) connected to a recording device such as EP Labsystem Pro Recording System (Boston Scientific, Boston MA). Common ventricular studies will include ventricular pacing from one or more sets of electrodes and analysis of sensed electrograms from adjacent sites.

### 3D Anatomical mapping

Electroanatomical mapping systems allow for spatially tracked EP studies with real-time visualization of cardiac geometry. Mapping catheters are a special type of EP catheters which can be tracked through space in a magnetic or electric field using a tool such as CARTO™ 3 (Biosense Webster, Irvine, CA), EnSite Precision (Abbott, Chicago, IL), or Rhythmia HDx (Boston Scientific, Boston, MA). Field-generating electrodes are placed beneath the thorax before surgical setup. For endocardial mapping, a catheter is introduced typically through the femoral vein or artery, and guided into a specific chamber of interest; epicardial mapping can be performed by guiding a catheter on the surgically exposed epicardium of the heart. An initial anatomic shell of the heart is created by roving the catheter along the surface of the heart. Once acquired, electroanatomic data such as voltage and local activation time (LAT) maps are obtained. Voltage maps assess myocardial integrity and scar burden, while LAT maps can be used to assess conduction velocity and patterns. Since anatomical mapping provides a global view of the heart, epicardial and endocardial mapping can be performed simultaneously, which is particularly useful in studies in the ventricles. Of note, use of 3D electroanatomical mapping on epicardial study does have some limitations. First, because the anterior electrode patch is displaced inferolaterally due to sternotomy, usual anatomical surfaces may appear skewed. Second, sheep have a very narrow and deep thorax with a posterior heart orientation when supine, which should be accounted for when rendering final images for publication. Nonetheless, 3D mapping has tremendous use in grasping the voltage map and LAT when describing conditions such as in scar related re-entry model.

## Limitations and surgical considerations

Surgical training is essential for performing a sternotomy, requiring the surgeon to maintain a balance of technical expertise and anatomical awareness. Surgeons must be thoroughly familiar with both human and veterinary anatomy to operate safely and effectively. A subtle but important consideration is the highly vascular hypodermis adipose tissue located along the mid-sternal line, which is encountered during the initial cutdown. Careful separation of the skin layers is necessary and should be accompanied by appropriate coagulation techniques to control bleeding. In large animal models such as sheep, the surgeon must also account for the non-linear structure of the sternum, requiring careful manual palpation and use of the sternal saw to avoid internal injury. Furthermore, while repeat sternotomies may be necessary for survival studies involving the longitudinal observation of injected materials or epicardial patches, they are often discouraged unless strictly required for the intervention. This is particularly true in ovine models, where the animal’s habitual sternal recumbency (resting on the chest) makes sternotomy recovery far more difficult than in porcine models [8].

From a safety and methodology standpoint, the minimally invasive percutaneous approach—such as balloon occlusion for post-MI VT models—is often preferred, as literature indicates it yields significantly higher survival rates (often exceeding 90%) compared to the physiological stress of an open-chest procedure [9]. One must also consider the inherent topographical differences of the target site; the epicardial surface is relatively smooth, lacking the complex, highly trabeculated “hills and cliffs” found within the endocardium. Finally, spatial data integrity remains a concern, as 3D electroanatomical mapping can be frequently skewed due to off-set al.ignment when transitioning between the surgical field and digital rendering, necessitating rigorous calibration to ensure translational accuracy.

## Impact on translational research

The translational relevance of surgical epicardial exposure in animal models is pivotal for bridging the gap between benchtop innovation and clinical cardiac therapy. By providing direct access to the epicardium, these surgical approaches facilitate the precise delivery and monitoring of cardiac regeneration strategies, including the engraftment of stem cell applications aimed at repairing infarcted myocardium [8]. This access is equally critical for the development and fixation of hydrogels and absorbable epicardial patches, which serve as scaffolds for tissue engineering or as localized drug-delivery systems [9, 10]. Furthermore, the epicardial interface allows for the seamless application of novel nanomaterials to the heart, enabling targeted interventions at the molecular level [6, 7, 11]. Beyond structural repair, the integration of specialized hydrogels for electrical sensing and other biocompatible fluids offers a sophisticated means of mapping cardiac electrophysiology with high fidelity, ensuring that next-generation diagnostics and therapeutics are both biologically integrated and functionally effective.

## Conclusions

Large animal EP studies enable future progress in the field of electrophysiology. In this article, we have shown a step-by-step method of how we commonly access the epicardial space to perform acute EP studies in a large animal sheep model. Epicardial access offers the advantages of direct visualization of the experimental site, facile catheter manipulation, direct access to epicardial coronary arteries, and the potential to test new methods and materials on the cardiac surface without the risks of charge dispersion, which is common on endocardial surfaces. We have used these techniques successfully to validate carbon nanotube fibers as a flexible electrically conductive suture to restore electrical conduction within the heart. Given the myriad uses of the surgical technique, we foresee its future use in a variety of surgical preclinical studies. Additionally, these methods could be used in the development of novel epicardial catheter techniques as well.

## Data Availability

No datasets were generated or analysed during the current study.

## Abbreviations and Acronyms

DD: Duodecapolar
EGM: Electrogram
EP: Electrophysiology
IACUC: Institutional Animal Care and Use Committee
RAA: Right atrial appendage
